# Supplementary material for: MetaRibo-Seq measures translation in microbiomes
Source: Nat Commun. 2020 Jun 29;11:3268. doi: 10.1038/s41467-020-17081-z (PMC7324362; doi:10.1038/s41467-020-17081-z)
Supplement: Supplementary file 10 — Supplementary Data 7 [file 41467_2020_17081_MOESM10_ESM.zip › File2/Confidence_High_Taxonomy/84865_out.krona.html]

Javascript must be enabled to view this page.

members
magnitude
magnitudeUnassigned
count
unassigned
taxon
rank

84865\_out

3

2
1
superkingdom

phylum
1224
1

1
28216
class

1
80840
order

1
80864
family

28065
1

SRS023914\_contig\_number\_28500
genus

superkingdom
2759
2

kingdom
4751
2

subkingdom
451864
2

phylum
4890
2

147538
2
subphylum

class
147548
1

order
5178
1

family
1
5181

genus
253308
1

species
253309
1

SRS013687\_contig\_number\_contig-100\_3385.181658

class
147550
1

1
222544
subclass

order
1
5151

5152
1
family

genus
360145
1

255215
1

SRS053356\_contig\_number\_44995
species
